# Diversity and Recombination of Dispersed Ribosomal DNA and Protein Coding Genes in Microsporidia

**DOI:** 10.1371/journal.pone.0055878

**Published:** 2013-02-06

**Authors:** Joseph Edward Ironside

**Affiliations:** Institute of Biological, Environmental and Rural Sciences, Aberystwyth University, Aberystwyth, United Kingdom; University of Ottawa, Canada

## Abstract

Microsporidian strains are usually classified on the basis of their ribosomal DNA (rDNA) sequences. Although rDNA occurs as multiple copies, in most non-microsporidian species copies within a genome occur as tandem arrays and are homogenised by concerted evolution. In contrast, microsporidian rDNA units are dispersed throughout the genome in some species, and on this basis are predicted to undergo reduced concerted evolution. Furthermore many microsporidian species appear to be asexual and should therefore exhibit reduced genetic diversity due to a lack of recombination. Here, DNA sequences are compared between microsporidia with different life cycles in order to determine the effects of concerted evolution and sexual reproduction upon the diversity of rDNA and protein coding genes. Comparisons of cloned rDNA sequences between microsporidia of the genus *Nosema* with different life cycles provide evidence of intragenomic variability coupled with strong purifying selection. This suggests a birth and death process of evolution. However, some concerted evolution is suggested by clustering of rDNA sequences within species. Variability of protein-coding sequences indicates that considerable intergenomic variation also occurs between microsporidian cells within a single host. Patterns of variation in microsporidian DNA sequences indicate that additional diversity is generated by intragenomic and/or intergenomic recombination between sequence variants. The discovery of intragenomic variability coupled with strong purifying selection in microsporidian rRNA sequences supports the hypothesis that concerted evolution is reduced when copies of a gene are dispersed rather than repeated tandemly. The presence of intragenomic variability also renders the use of rDNA sequences for barcoding microsporidia questionable. Evidence of recombination in the single-copy genes of putatively asexual microsporidia suggests that these species may undergo cryptic sexual reproduction, a possibility with profound implications for the evolution of virulence, host range and drug resistance in these species.

## Introduction

Microsporidia are near-ubiquitous intracellular parasites of animals and protists. They are closely related to fungi, although their taxonomic status (as a clade within the fungi or a sister clade to the fungi) remains the subject of debate [1], [2]. Microsporidia possess the smallest genomes of any eukaryotic organisms and cause a variety of important medical, veterinary, agricultural and ecological impacts [3]. Many studies of microsporidia and other parasites have attempted to classify strains by amplifying and sequencing variable regions of the genome such as the ribosomal internal transcribed spacer region (ITS) [4], [5], [6], [7], [8]. The ITS is also proposed as a universal DNA barcode marker for fungi [9]. However, given the reduced and rearranged nature of microsporidian ribosomal DNA (described below), “universal” fungal primers are unlikely to amplify the microsporidian ITS reliably. The dispersed nature of rDNA repeats in some microsporidian species [10] also calls into question the assumption that repeats will be homogenised by concerted evolution.

Genes coding for ribosomal RNA subunits usually occur as multiple copies within eukaryotic genomes. They are typically organised into rDNA units, each consisting of 18S, 23S and 5.8S subunits, separated by two internal transcribed spacer regions (ITS1 and ITS2). Within most eukaryotic genomes, rRNA genes exist as tandem arrays, with each rDNA unit separated by an intergenic spacer (IGS). The units of these tandem arrays are subject to homogenization by unequal crossing over and gene conversion [11], a process known as concerted evolution. A result of concerted evolution is that RNA units at different positions within the genome of a species tend to be more similar than RNA units at equivalent positions in the genomes of different species.

However, in rare cases, rDNA units are dispersed throughout the genome rather than organised in tandem arrays. Because unequal crossing over and gene conversion occur less frequently between sequences on heterologous chromosomes than on homologous chromosomes [12], concerted evolution is predicted to act less strongly upon dispersed rDNA units than upon tandem repeats. This prediction is supported by the finding that sequences belonging to the dispersed rDNA units of Apicomplexa often cluster phylogenetically between species rather than within species [13]. This indicates that concerted evolution is insufficient to homogenise these dispersed units and suggests that their evolution results instead from a birth and death process in which new copies are repeatedly produced by gene duplication and removed by gene deletion [14]. A similar process appears to underlie the evolution of dispersed 5S rRNA units in fungi [15], plants [16] and animals [17]. Dispersed 5S rDNA units also appear to evolve through a combination of concerted and birth-and-death processes in fish [18] and in the mussel *Mytilus* [19]. Intragenomic variation of rDNA can cause errors when rDNA is used to discriminate strains or species, as in the case of the putative apicomplexan species *Eimeria mitis* and *Eimeria mivati* [20].

Within *Nosema*, a genus of microsporidian parasites, one species *N. apis* possesses tandemly repeated rDNA units [21] while in another species *N. bombycis*, rDNA units are dispersed over multiple chromosomes [10]. Furthermore, within isolates of *Nosema bombycis* from the host *Bombyx mori*, rDNA units are highly variable, differing with regard to nucleotide sequence, subunit organisation and the presence of transposable elements [22]. This suggests that, in *Nosema*, the evolution of rDNA units may occur through a birth-and-death process rather than through concerted evolution.

Some, but not all, *Nosema* species also possess an unusual “reversed” arrangement of rDNA subunits [23], [24] in which the large 18S subunit occurs upstream of the smaller 16S subunit. The two subunits are separated by a spacer region, described by Huang et al. [23] as an internal transcribed spacer (ITS), although no evidence of transcription is presented. Downstream of the 16S subunit is a small, 5S subunit, separated from the 16S subunit by a second spacer region, described by Huang et al. [23] as an intergenic spacer (IGS). Other *Nosema* species possess an arrangement of the rDNA unit more common among microsporidia, with the 16S subunit positioned upstream of the 18S subunit and separated from it by a short ITS region. Intragenomic variation in the ITS of *N. bombi* [25], [26] indicates that these species may also experience relaxed convergent evolution, despite evidence that the rDNA unit is repeated tandemly [21]. In *N. ceranae*, a 5S subunit occurs upstream of the 16S subunit, separated from it by an IGS [8]. The presence of two different subunit arrangements in different species of the same genus suggests a birth and death process in which a mutation of the subunit arrangement in one rDNA unit spread to other locations in the genome concurrently with the extinction of the original arrangement [24].

Species within the genus *Nosema* also vary markedly in their transmission strategies and life cycles [24]. While some species are transmitted vertically from female hosts to their offspring, others are transmitted horizontally via infectious spores and a third group combine these two modes of transmission [24]. Most *Nosema* species produce diplokaryotic stages that develop in direct contact with the host cell’s cytoplasm but some species also produce unikaryotic stages that develop within a sporophorous vesicle, usually in groups of eight [27], [28]. Such species were fomerly allocated to the genus *Vairimorpha* but this is now acknowledged to be synonymous with *Nosema* [29]. The unikaryotic phase is thought to be associated with sexual reproduction and species lacking this phase are often assumed to be asexual [24]. By affecting the degree to which parasite populations mix, these differences in life cycle and transmission are predicted to have important implications for the degree of intragenomic and intergenomic diversity found in *Nosema* parasites within a single host.

For the purposes of this study, *Nosema* species were divided into three groups (Table 1), depending on their life cycles. Group 1 contains species which lack the putatively sexual unikaryotic phase and the ability to undergo horizontal transmission. Because all transmission between hosts is vertical, typically involving approximately 200 parasite cells, a bottleneck in population size occurs each host generation [30], [31]. It is therefore predicted that the genetic diversity of group 1 species should be eroded rapidly, resulting in intergenomic near-homogeneity within any given host. Most diversity observed in multicopy regions of DNA should therefore represent intragenomic variation between copies. Group 2 contains species which are capable of horizontal transmission (with or without supplementary vertical transmission) but lack a unikaryotic phase in their life cycle and are presumed to be asexual. Mixing of cell lineages through coinfection is likely to occur in Group 2 species and so intergenomic diversity within hosts is predicted to be higher than in species of Group 1. Group 3 contain species that are horizontally transmitted and have lifecycles containing a unikaryotic phase, suggesting that they undergo meiosis and sexual reproduction. As sexual, horizontally transmitted species Group 3 species are expected to show higher levels of recombination than species of groups 1 or 2.

**Table 1:** Assignment of *Nosema* species to life cycle groups based on transmission and sexuality.

Group	Parasite	Host(s)
1: No unikaryotic phase (putatively asexual). No horizontal transmission.	*Nosema granulosis*	*Gammarus duebeni*
2: No unikaryotic phase (putatively asexual). Horizontal transmission.	*Nosema bombycis*	*Bombyx mori*
		*Trichoplusia ni*
		*Tyria jacobaeae*
	*Nosema apis*	*Apis mellifera*
	*Nosema lymantriae*	*Lymantria dispar*
3. Unikaryotic phase (putatively sexual). Horizontal transmission.	*Vairimorpha cheracis*	*Cherax destructor*
	*Vairimorpha disparis*	*Lymantria dispar*
	*Vairimorpha necatrix*	*Lacanobia oleracea*

In this study, the genetic diversity of *Nosema* species belonging to the three groups was examined by comparing the genetic diversity of the protein-coding genes *RNA Polymerase II* (*RPB1*), *Elongation Factor-1 alpha* (*EF-1α*) and *Surface antigen protein 30.4* (*SAP30.4*) between these species. The process of evolution of rDNA units in various *Nosema* species was then investigated by analysing the pattern of diversity of rDNA sequences within and between isolates from different geographical locations. If concerted evolution occurs then the diversity of rDNA sequences should be lower in Group 1 species than in horizontally transmitted Group 2 or Group 3 species. Furthermore, rDNA diversity should be structured primarily between species and between populations within species rather than between copies within the genome. Additionally, if the species lacking a unikaryotic spore cycle are truly asexual, then Group 3 species should demonstrate evidence of meiotic recombination in single copy genes while species of groups 1 and 2 should not.

## Results

### rDNA Sequences

**Figure 1:**
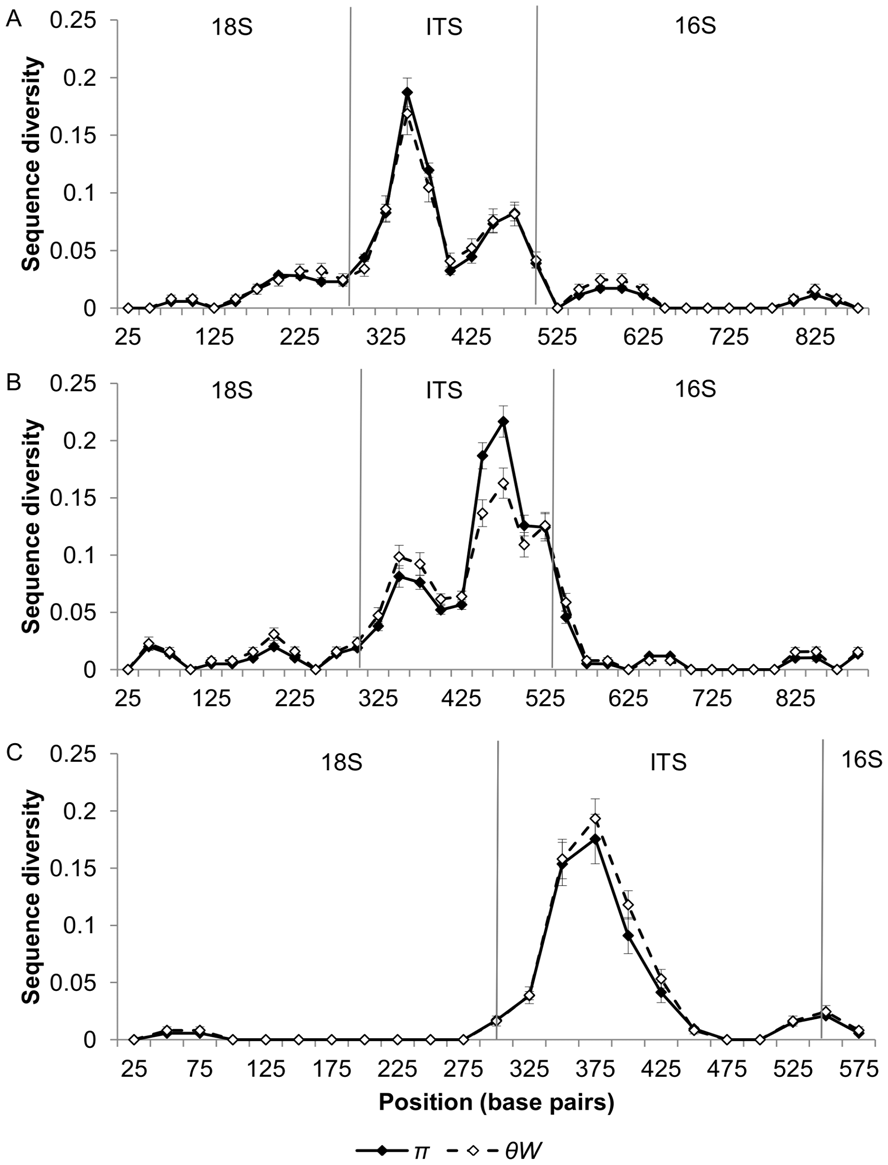
Sliding window analysis of nucleotide diversity in the ITS and flanking 18S and 16S rDNA regions of *N. bombycis* (Part A), *N. granulosis* (Part B) and *V. cheracis* (Part C). A sliding window of 50 base pairs is used, with an increment of 25 base pairs. Nucleotide diversity is calculated as the average heterozygosity per site (*π*) and the average number of nucleotide differences per site (*θ_W_*). Error bars show the standard error for each window.

**Figure 2:**
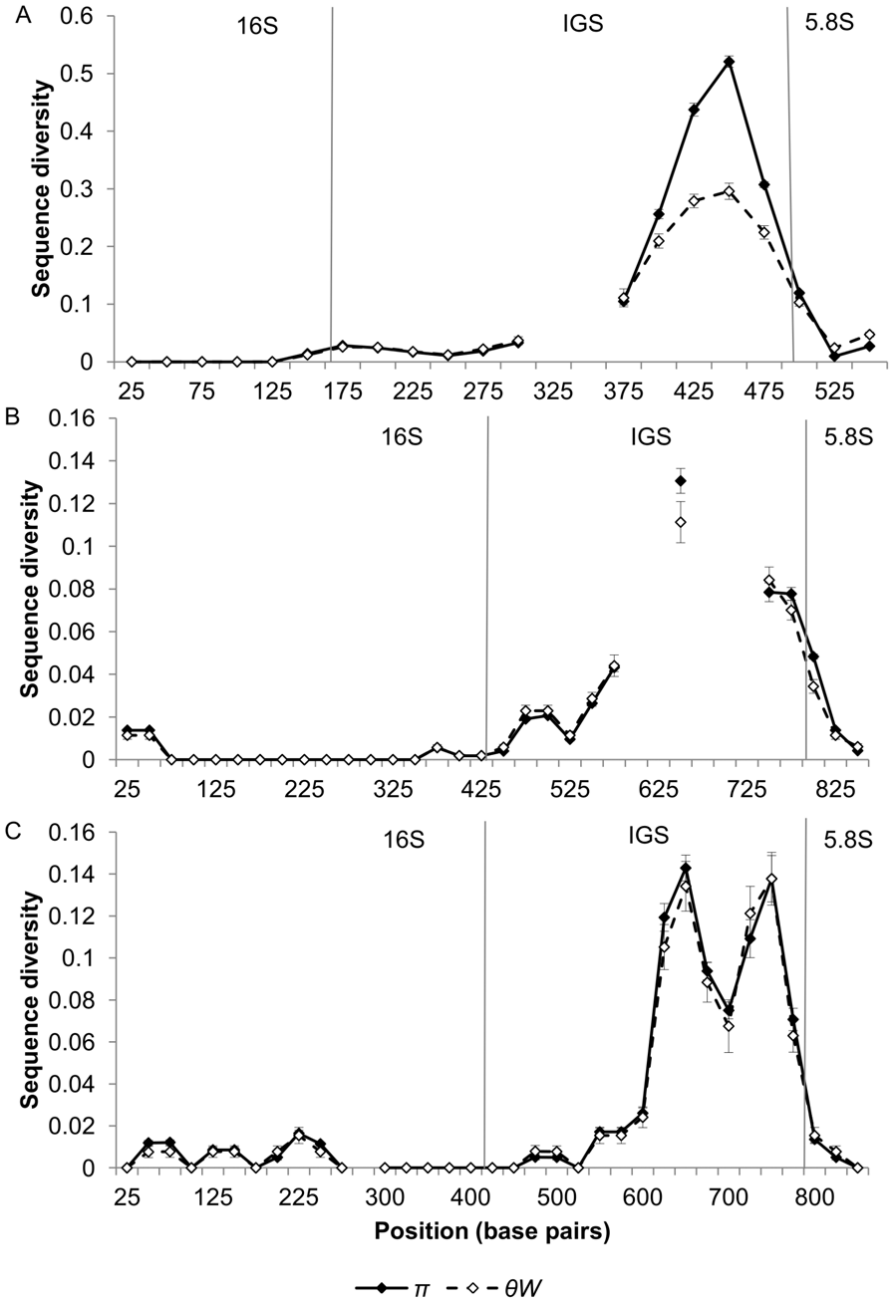
Sliding window analysis of nucleotide diversity in the IGS and flanking 16S and 5.8S rDNA regions of *N. bombycis* (Part A), *N. granulosis* (Part B) and *V. cheracis* (Part C). A sliding window of 50 base pairs is used, with an increment of 25 base pairs. Nucleotide diversity is calculated as the average heterozygosity per site (*π*) and the average number of nucleotide differences per site (*θ_W_*). Error bars show the standard error for each window. Regions in which sequences could not be aligned due to multiple insertions and deletions are indicated by missing data (breaks in the line).

**Figure 3:**
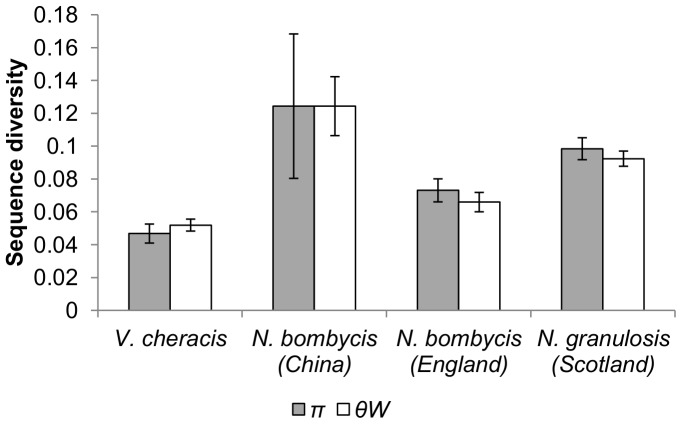
Nucleotide diversity of the ITS and flanking 18S and 16S rDNA regions of *N. bombycis*, *N. granulosis* and *V. cheracis* isolates. Nucleotide diversity is calculated as the average heterozygosity per site (*π*) and the average number of nucleotide differences per site (*θ_W_*). Error bars show the standard error for each isolate.

**Figure 4:**
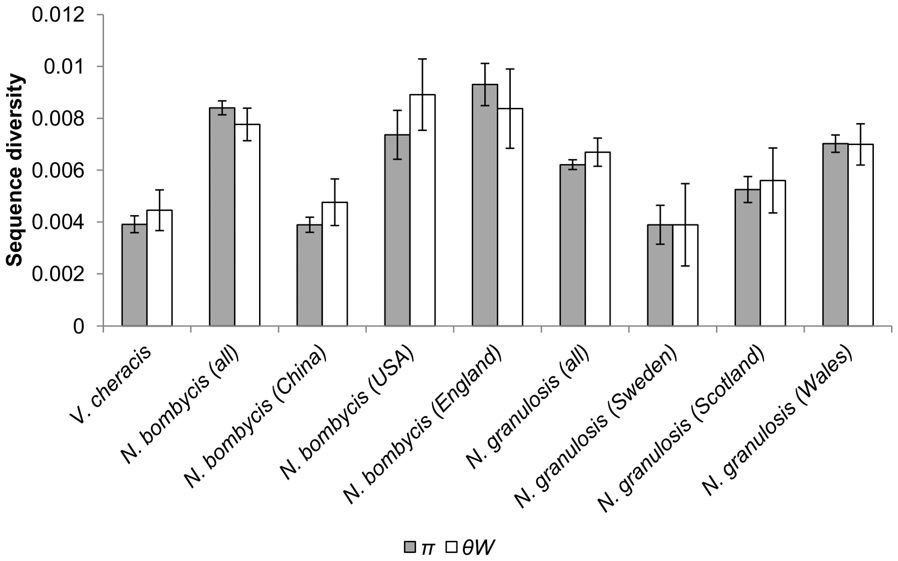
Nucleotide diversity of the IGS and flanking 16S and 5.8S rDNA regions of *N. bombycis*, *N. granulosis* and *V. cheracis* isolates. Nucleotide diversity is calculated as the average heterozygosity per site (*π*) and the average number of nucleotide differences per site (*θ_W_*). Error bars show the standard error for each isolate.

The diversity of cloned ribosomal DNA sequences appears similar in *N. granulosis* (group 1), *N. bombycis* (group 2) and *V. cheracis* (group 3). In all three species, sliding window analysis of cloned rDNA sequences (Genbank JX213695–213745, JX213774–213781) indicates that the ITS (Figure 1) and IGS (Figure 2) spacer regions of the rDNA repeat unit have elevated nucleotide diversity when compared with the flanking 18S, 16S and 5S rRNA genes. This indicates that purifying selection eliminates mutations in the rRNA genes but is relaxed in the spacer regions. There is no significant difference in ITS diversity between isolates of *N. bombycis* (Figure 3) and no significant differences in IGS diversity between isolates of *N. bombycis* or *N. granulosis* (Figure 4). In each of these cases, the diversity of pooled sequences from all isolates is not significantly greater than that of individual isolates, indicating that most genetic diversity in rDNA sequences occurs within isolates rather than between isolates.

**Figure 5:**
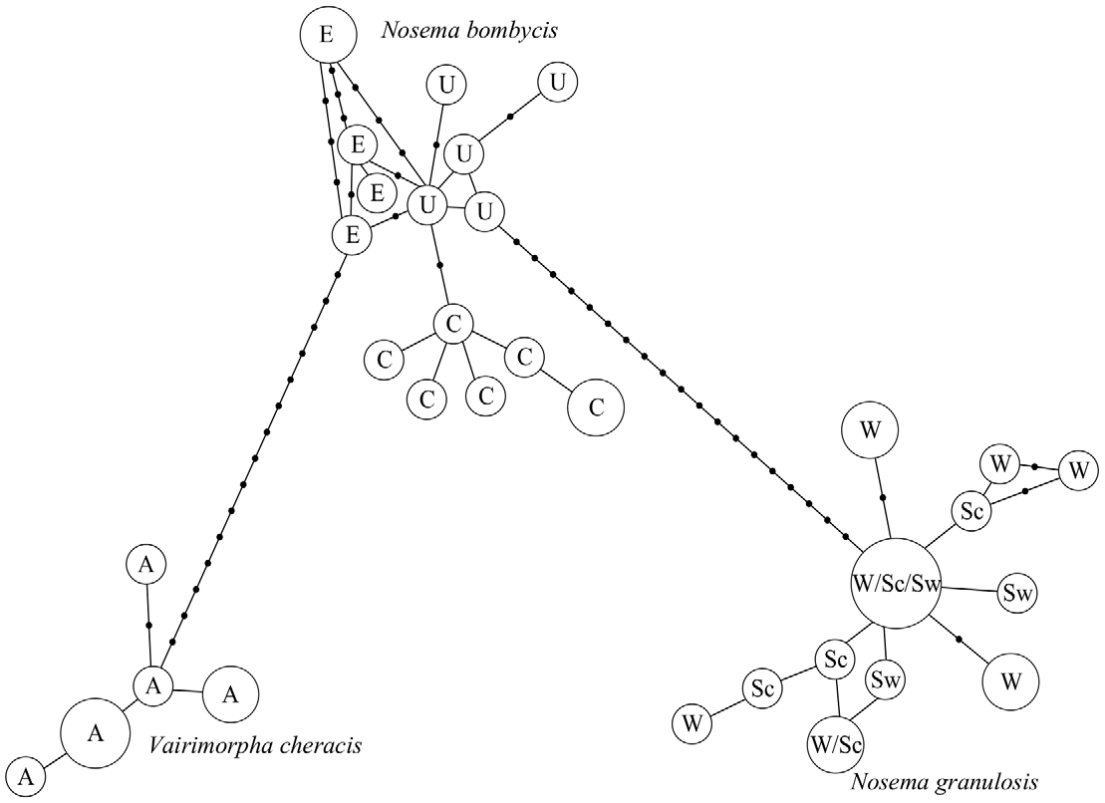
Haplotype network of cloned rDNA sequences containing the IGS and flanking 16S and 5.8S rDNA regions of *N. bombycis*, *N. granulosis* and *V. cheracis* isolates. Branch lengths are proportional to the number of mutations separating haplotypes. Areas of circles are proportional to the number of clones containing each haplotype. Isolates are labelled as follows: A = *V. cheracis* (Australia), E = *N. bombycis* (England), U = *N. bombycis* (USA), C = *N. bombycis* (China), W = *N. granulosis* (Wales), Sc = *N. granulosis* (Scotland), Sw = *N. granulosis* (Sweden).

Haplotype networks indicate that rDNA haplotypes of *N. bombycis*, *N. granulosis* and *V. cheracis* fall into distinct clusters (Figure 5). While IGS sequences of the group 1 species *N. granulosis* do not cluster within isolates, the rDNA sequences of the group 2 species *N. bombycis* show clustering within isolates (Figure 5). These results are confirmed by permutation tests of F_ST_ which indicate significant population structure between species and among isolates of *N. bombycis* but not among isolates of *N. granulosis* (Table 2). Recombination events were detected through analysis of cloned ITS and IGS sequences in all three species (Table 3).

**Table 2:** Population structure of ribosomal IGS sequences from *N. bombycis*, *N.granulosis* and *V. cheracis* isolates.

			*F_ST_*				
Isolate	Sc	W	Sw	C	U	E	A
Sc		0.02	0.10	0.92***	0.89***	0.88***	0.93***
W	0.00013		0.05	0.90***	0.87***	0.87***	0.91***
Sw	0.00049	0.0003		0.93***	0.90***	0.89***	0.94***
C	0.05296	0.05207	0.05266		0.31**	0.45***	0.91***
U	0.05049	0.04961	0.0502	0.00258		0.17**	0.87***
E	0.05382	0.05294	0.05353	0.00552	0.00176		0.85***
A	0.05903	0.05938	0.0605	0.04216	0.03901	0.03771	
			*D_a_*				

Comparisons were made using Wright’s index of fixation (*F_ST_*) and net nucleotide substitutions per isolate (*D_a_*). Levels of significance: *0.05, **0.01, ***0.001. Isolates are labelled as follows: A = *V. cheracis* (Australia), E = *N. bombycis* (England), U = *N. bombycis* (USA), C = *N. bombycis* (China), W = *N. granulosis* (Wales), Sc = *N. granulosis* (Scotland), Sw = *N. granulosis* (Sweden).

**Table 3:** Recombination events detected in alignments of cloned sequences using RDP4.

Gene	Species	Break points	Recombinant sequences	Parental sequences	Methods	p
IGS	*N. granulosis*	561–673	Wales_Frd9, Wales_Frd11	Sweden_Nyd17, Wales_Frd4	3Seq	0.004
		487–630	Sweden_Nyd6	Wales_Frd10, Scotland_Jsb5	SiScan	0.048
		604-?	Scotland_Jsb5	Wales_Frd12, unknown	SiScan	0.021
		?-677	Sweden_Nyd8	Wales_Frd10, unknown	SiScan	0.019
	*V. cheracis*	169–255	Vch8	Vch2, Vch7	MaxChi, SiScan, 3Seq	0.001
		?-221	Vch6	Vch2, Vch1	MaxChi	0.009
ITS	*N. bombycis*	359–452	China_Bm8	England_Nty8, unknown	GeneConv, BootScan, MaxChi, Chimaera, 3Seq	0.019
Rpb1	*N. bombycis*	2421–2648	China_Bm9, England_Tj9	England_Tj5, USA_Tn5	SiScan	0.012
		484-?	China_Bm9	England_Tj5, unknown	MaxChi, 3Seq	0.018
	*V. necatrix*	446-?	Vnec3	Vnec1, Vnec2	RDP, GeneConv,BootScan, 3Seq	0.048

Only recombination detection methods providing statistically significant support for a given recombination event are listed. P-values are for Bonferroni-corrected multiple comparisons.

Sequence diversity within the short ITS regions of *N. apis* (group 1) and *V. necatrix* (group 2) does not appear to be elevated compared to the flanking 18S and 16S regions while analysis of the cloned ribosomal sequences (Genbank JX213654–213662, JX213789-JX213795) provides some evidence of recombination (Table 3).

### Protein-coding Sequences

**Figure 6:**
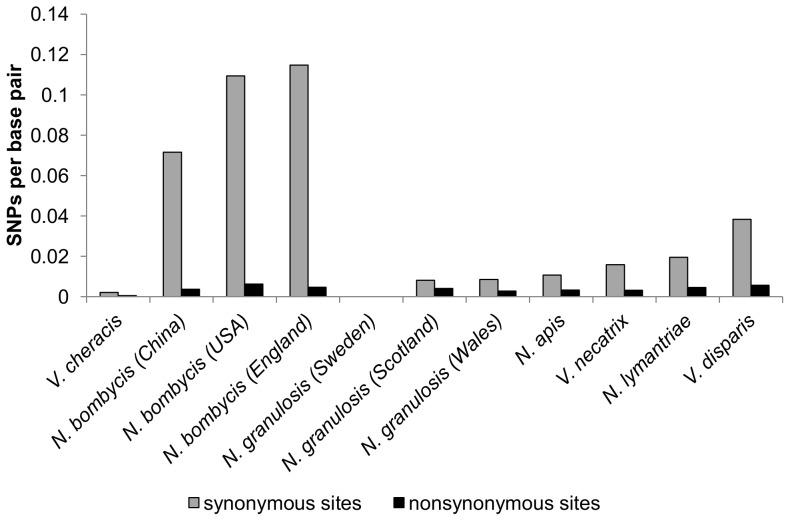
Numbers of synonymous and nonsynonymous single nucleotide polymorphisms (SNPs) detected by direct sequencing of the *RPB1* gene from *Nosema* isolates.

Direct sequencing of fragments of the largest subunit of *RNA Polymerase II* (*RPB1*) amplified from *N. granulosis*, *V. cheracis* and *N. apis* (Genbank JX213746–213748, DQ996235, DQ996230) revealed little genetic diversity within isolates (Table 4, Figure 6). However, fixed differences indicated genetic divergence between the two British isolates of *N. granulosis* (from Wales and Scotland) and the Swedish isolate (Table 5). *RPB1* sequences from *V. necatrix*, *V. disparis* and *N. lymantriae* (Genbank DQ996236, JX213748, JX213749) exhibited levels of diversity slightly higher than those of *N. granulosis* (Table 4). In contrast, *RPB1* sequences from *N. bombycis* (Genbank DQ996231) displayed extremely high levels of synonymous nucleotide diversity. Many polymorphisms are shared between *N. bombycis* isolates (Table 6) and there are relatively few fixed differences between isolates. Direct sequencing revealed no indels in *RPB1* in any species and the vast majority of single nucleotide polymorphisms occurred at third codon positions (Table 4, Figure 6). This indicates strong purifying selection to maintain gene function, confirming that the amplified fragments belong to functional genes. Analysis of six cloned *RPB1* sequences (Genbank JX213750–JX213755, JX213663–JX213670, JX213796–JX213798) indicated recombination events in *N. bombycis* and *V. necatrix* but provided no evidence of recombination in *N. apis* (Table 3).

**Table 4:** Synonymous and nonsynonymous substitutions detected by direct sequencing of protein-coding genes from *Nosema* isolates.

Gene	Isolate	Length (bp)	Synonymous substitutions	Nonsynonymous substitutions	*Ka*/*Ks*
*EF-1α*	*N. bombycis* (China)	948	15	0	0
	*N. bombycis* (USA)	948	13	0	0
	*N. bombycis* (England)	948	14	0	0
*Sap 30.4*	*N. bombycis* (China)	714	10	3	0.15
	*N. bombycis* (USA)	686	13	2	0.08
	*N. bombycis* (England)	736	13	2	0.08
*RPB1*	*N. bombycis* (China)	2851	68	7	0.05
	*N. bombycis* (USA)	2851	104	12	0.06
	*N. bombycis* (England)	2851	109	9	0.04
	*N. granulosis* (Sweden)	881	0	0	0
	*N. granulosis* (Wales)	1104	3	3	0.5
	*N. granulosis* (Scotland)	1055	3	2	0.33
	*V. cheracis*	2811	2	1	0.25
	*N. apis*	2183	8	5	0.31
	*V. necatrix*	3865	20	8	0.2
	*N. lymantriae*	2838	19	9	0.24
	*V. disparis*	2838	40	10	0.125

**Table 5:** Pairwise comparisons of *RPB1* sequences between *N. granulosis* isolates, showing frequency of shared polymorphisms, unique polymorphisms and fixed differences.

		Shared/unique polymorphisms	
	Scotland	Wales	Sweden
Scotland		4/3	0/0
Wales	0		0/0
Sweden	12	12	
		Fixed differences	

**Table 6:** Pairwise comparisons of *RPB1* sequences between *N. bombycis* isolates, showing frequency of shared polymorphisms, unique polymorphisms and fixed differences.

		Shared/unique polymorphisms	
	China	USA	England
China		31/130	39/115
USA	3		58/119
England	13	11	
		Fixed differences	

**Figure 7:**
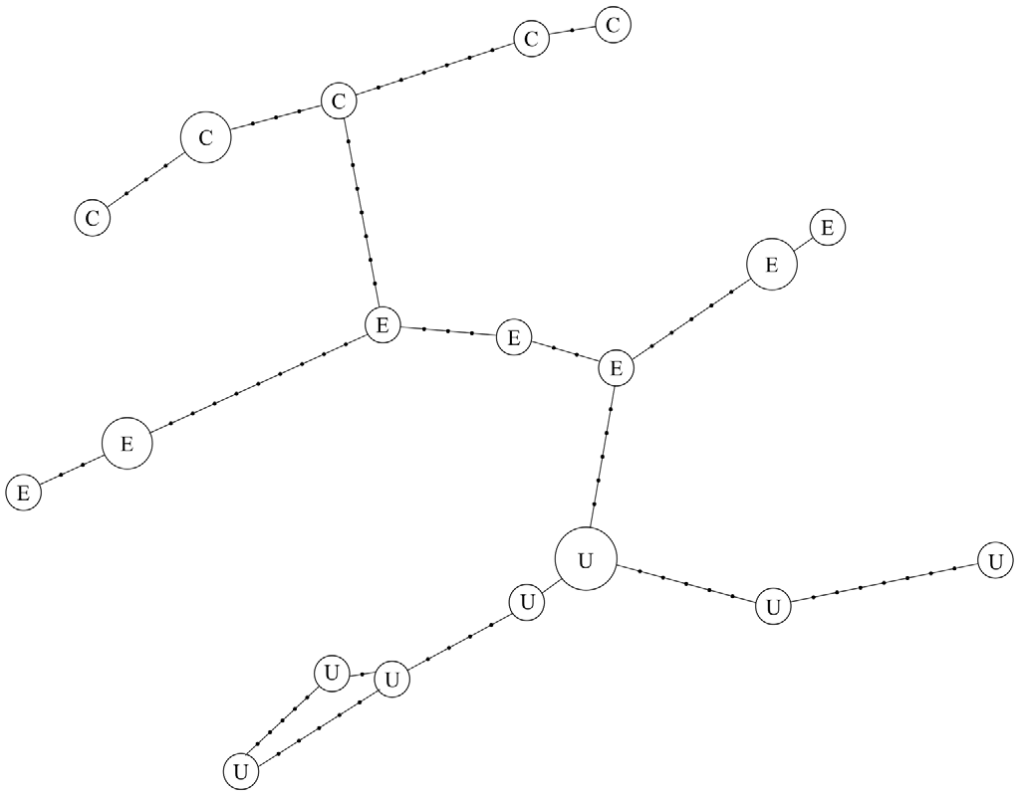
Haplotype network of cloned *EF-1α* sequences from *N. bombycis* isolates. Branch lengths are proportional to the number of mutations separating haplotypes. Areas of circles are proportional to the number of clones containing each haplotype. Isolates are labelled as follows: E = England, U = USA, C = China.

Fragments of *EF-1α* and *SAP 30.4* amplified from *N. bombycis* (Genbank JX213671–JX213694, JX213799, JX213756–JX213773) display moderate levels of synonymous nucleotide diversity (Table 7). Single nucleotide polymorphisms occur in both genes within all three isolates, mainly at third codon positions (Table 4). Direct sequencing of *EF-1α* revealed a single, polymorphic indel within the isolate from the USA. This is 391 bp in length and is located within a spliceosomal intron. Haplotype networks indicate strong clustering of *EF-1α* by isolate, but only weak clustering of *SAP 30.4* (Figure 7). However, permutation tests of F_ST_, indicate restricted gene flow between isolates in both *EF-1α* (F_ST_ = 0.38, P<0.0001) and *SAP 30.4* (F_ST_ = 0.20, P<0.05). Analysis of cloned sequences provides no evidence for multiple recombination events in either of these genes (Table 3).

**Table 7:** Genetic diversity in cloned sequences from *Nosema* samples.

Sequence	Species	N	Length (bp)	*π*±SD	*θ_W_*±SD
18S-ITS-16S	*N. bombycis* (all)	7	899	0.0230±0.01314	0.0235±0.00333
	*N. bombycis* (China)	2	899	0.0288±0.02650	0.0265±0.00553
	*N. bombycis* (England)	5	899	0.0224±0.01273	0.0210±0.00341
	*N. granulosis*(Scotland)	8	944	0.0320±0.01738	0.0318±0.00373
	*V. cheracis*	7	604	0.0204±0.01258	0.0225±0.00398
16S-ITS-18S	*N. apis*	9	782	0.0053±0.00384	0.0072±0.00185
	*V. necatrix*	7	795	0.0029±0.00172	0.0031±0.00126
16S-IGS-5S	*N. bombycis* (all)	17	617	0.0718±0.02588	0.0515±0.00583
	*N. bombycis* (China)	7	617	0.0557±0.02494	0.0446±0.00638
	*N. bombycis* (USA)	5	617	0.0741±0.03504	0.0579±0.00787
	*N. bombycis* (England)	5	617	0.0888±0.04282	0.0707±0.00870
	*N. granulosis*(all)	19	1022	0.0161±0.00740	0.0148±0.00241
	*N. granulosis*(Scotland)	5	1022	0.0180±0.01031	0.0170±0.00334
	*N. granulosis*(Wales)	11	1022	0.0167±0.00778	0.0149±0.00263
	*N. granulosis*(Sweden)	3	1022	0.0100±0.00746	0.0100±0.00302
*EF-1α*	*N. bombycis* (all)	24	923	0.01302±0.00069	0.01134±0.00182
	*N. bombycis* (China)	6	923	0.00803±0.00173	0.00713±0.00184
	*N. bombycis* (USA)	9	923	0.00838±0.00174	0.00798±0.00178
	*N. bombycis* (England)	9	923	0.01095±0.00131	0.00837±0.00183
*Sap 30.4*	*N. bombycis* (all)	18	736	0.01076±0.00076	0.00751±0.00172
	*N. bombycis* (China)	5	736	0.01005±0.00239	0.00913±0.00244
	*N. bombycis* (USA)	5	736	0.00842±0.00247	0.00783±0.00226
	*N. bombycis* (England)	8	736	0.00907±0.0011	0.00629±0.00182
*RPB1*	*N. bombycis* (all)	6	2646	0.03132±0.00322	0.03161±0.00229
	*N. apis*	7	1571	0.00029±0.0002	0.00041±0.00041
	*V. necatrix*	3	1056	0.00631±0.00202	0.00631±0.002

Nucleotide diversity is calculated as the average heterozygosity per site (*π*) and the average number of nucleotide differences per site (*θ_W_*).

## Discussion

The findings of this study provide evidence for intergenomic and intragenomic diversity of rDNA and protein coding sequences within isolates of microsporidia acquired from single hosts. Very high levels of diversity are present in the rDNA intergenic spacer regions and internal transcribed spacer regions of *V. cheracis*, *N. bombycis* and *N. granulosis*, including evidence of multiple insertion, deletion and substitution events. In contrast, the functional rDNA subunits are relatively conserved. This suggests that the similarity of microsporidian rRNA gene products is maintained by purifying selection rather than by concerted evolution [14]. High levels of diversity also occur within the sequences of the protein-coding genes *RPB1*, *EF-1α* and *Sap30.4*. Again, the occurrence of the vast majority of polymorphic sites at synonymous positions suggests that the high rates of DNA sequence evolution known to occur in microsporidia [32], [33] are countered by strong purifying selection.

Sequence diversity of the rDNA IGS and ITS of the Group 1 species *N. granulosis* is similar to those of *N. bombycis* (Group 2) and *V. cheracis* (Group 3). Given that *N. granulosis* lacks horizontal transmission, its intergenomic diversity should be reduced due to repeated bottlenecks during vertical transmission. The similarity of rDNA diversity between species from groups 1, 2 and 3 therefore suggests that this diversity is primarily intragenomic.

Although most rDNA diversity occurs within, rather than between isolates, rDNA haplotypes form clusters within each of the three microsporidian species. This suggests that the length of evolutionary time since these species diverged has been sufficient to obscure shared histories of rDNA copies between species through slow concerted evolution and/or cumulative birth and death events. rDNA haplotypes also cluster within isolates of *N. bombycis*. However, rDNA haplotypes do not cluster within isolates of *N. granulosis*. This may reflect the fact that the *N. bombycis* isolates were collected from three different host species on three different continents while the *N. granulosis* isolates were collected from a single host species in north-west Europe.

The presence of more than two distinct haplotypes of the genes *EF-1α* and *Sap30.4* within *N. bombycis* isolates indicates that this diversity is not simply due to heterozygosity within diploid cells, but is likely to be due to intergenomic diversity. Synonymous diversity of *RPB1* within isolates of *V. cheracis*, *V. necatrix, N. apis, N. lymantriae* and *V. disparis* is much lower than that of *N. bombycis* and is more similar to that of *N. granulosis* despite the fact that the former five species are horizontally transmitted. In contrast, the diversity of *RPB1* at non-synonymous sites in *N. bombycis* is similar to those of the other *Nosema* species, producing values of *Ka/Ks* that are an order of magnitude lower in *N. bombycis* than in the other species.

The unusually high diversity of *RPB1* suggests that it may occur as multiple copies in *N. bombycis*. However, the genes *EF-1α* and *Sap30.4* also have high synonymous nucleotide diversity in *N. bombycis*, suggesting that a whole genome duplication in *N. bombycis* followed by divergence of paralogous copies may have increased the intragenomic diversity of all three genes. In support of this hypothesis, the estimated genome size of *N. bombycis* (15.33 Mbp) is greater than those published for other *Nosema* species [34], [35], [36], [37], all of which fall in the range 9.25–10.56 Mbp. However, in this case alleles would be expected to cluster into two divergent groups, present in all isolates and with little evidence of recombination between them. This does not appear to be the case for *RPB1*, *EF-1α* or *Sap30.4*. It therefore seems more likely that the higher *RPB1* diversity observed in *N. bombycis* is intergenomic, resulting from a more diverse population of parasites within each host than is found in the other *Nosema* species.

Recombination was detected between rDNA sequences in *N. granulosis*, *N. bombycis* and *V. cheracis*. Recombination was also detected in the protein-coding gene *RPB1* in *N. bombycis* and *V. necatrix*. This is surprising given the lack of unikaryotic stages in the life cycles of *N. granulosis* and *N. bombycis*, previously taken to indicate a lack of meiosis. Recombination between homologous and/or non-homologous copies of the rDNA unit within each genome may have occurred at mitosis. This possibility is suggested by studies of other apomictic species, which have revealed highly homogeneous rDNA arrays, indicating that mitotic recombination is sufficient for concerted evolution to occur in the absence of sex [38], [39]. However, it does not explain the evidence for multiple recombination events in *RPB1* in *N. bombycis* unless this gene occurs as multiple copies.

Alternatively, *N. bombycis* and *N. granulosis* may possess cryptic meiotic stages, making them capable of sexual reproduction. Unikaryotic cells have occasionally been observed in *N. bombycis* [40] while recombination between rDNA units has been detected in the honeybee pathogen *Nosema ceranae* [41] and is also suggested as an explanation for incongruous 16S and 18S rDNA phylogenies in a *Nosema* species from the host *Pieris rapae* [42]. The potential for *Nosema* strains to exchange genes through sexual reproduction has important implications for the evolution of virulence and drug resistance in these damaging pathogens.

In *Nosema* species possessing the rearranged version of the rDNA repeat unit (*V. cheracis*, *N. bombycis* and *N. granulosis* in this study), evidence for intragenomic diversity suggests that rDNA spacer regions are unsuitable for typing of strains. Evidence of intergenomic diversity also suggests that coinfection with multiple strains and recombination between strains is common. However, the high level of diversity found at synonymous sites in protein-coding genes such as *RPB1* suggest that these may be of use in strain typing. In the case of *N. granulosis*, for example, British and Swedish isolates could not be distinguished through analysis of the ribosomal IGS but could be distinguished clearly by comparing *RPB1* sequences.

In *Nosema* species possessing the non-rearranged version of the rDNA repeat unit (*N. apis, V. necatrix, N. lymantriae* and *V. disparis* in this study). The ITS region is very short (33–34 bp) and does not appear to be substantially more variable within isolates than the flanking rRNA subunit genes. This may be taken as evidence that the arrangement of rDNA units in tandem repeats has led to stronger concerted evolution in these species than in species possessing the rearranged rDNA unit. However, diversity within single hosts has been detected in the ITS of *N. bombi* [25], [43] and in intergenic regions flanking the rDNA units of *N. apis* [21] and *N. ceranae* [41].

In conclusion, the findings of this study provide some evidence for concerted evolution in the dispersed rDNA units of microsporidia. This is sufficient to produce clustering of rDNA copies within species, and even within populations, but is insufficient to prevent diversification of rDNA copies within microsporidian genomes. Additional genetic variation is provided by intergenomic diversity of microsporidia within single hosts and by recombination between the resulting heterogeneous strains. However, microsporidia appear to be subject to strong purifying selection, with the result that most genetic diversity occurs within rDNA spacer regions or at synonymous sites in protein-coding genes.

## Materials and Methods

### Samples

Three isolates of *N. bombycis* spores were obtained from the hosts *Bombyx morii* (China), *Trichoplusia ni* (USA) and *Tyria jacobaeae* (England). The Chinese isolate originated in cultured silk moths, the American isolate was purchased from the American Type Culture Collection (strain ATCC 30702) and the English isolate was obtained from a wild cinnabar moth population near Plymouth. The American isolate was originally described as *N. trichoplusiae* [44]. However, *N. bombycis* and *N. trichoplusiae* have since been shown to be synonymous [45]. Three isolates of *N. granulosis* were obtained from wild populations of the amphipod crustacean *Gammarus duebeni* from Anglesey (Wales, UK), Isle of Cumbrae (Scotland, UK) and Nynashamn (Sweden). Field studies at Plymouth, Nynashamn, Anglesey and Isle of Cumbrae did not involve protected species and were undertaken on public land that was not subject to any form of protection. No specific permits were required for the described field studies.

A single isolate of the putatively sexual, octosporous species *Vairimorpha cheracis*, originally obtained from a population of the Australian yabby *Cherax destructor* was donated by the University of New England, Australia. An isolate of *N. apis* was obtrained from honey bees (*Apis mellifera*) in Ireland and an isolate of *V. necatrix* was obtained from a laboratory population of the moth *Lacanobia oleracea* maintained at Central Science Laboratories, England. Isolates of *Vairimorpha disparis* and *Nosema lymantriae* from a laboratory population of Gypsy Moth *Lymantria dispar*, were donated by Illinois Natural History Survey, USA and used with their permission.

Each isolate was obtained from a single host individual and dissected using flame-sterilised forceps to avoid cross-contamination. The only exception to this was the case of *N. apis* in which spores were harvested from several honey bees from the same colony. Spores of *N. bombycis* (China and USA), *Vairimopha disparis* and *Nosema lymantriae* were purified using a percol gradient. Other isolates were not purified. Total DNA (containing host and microsporidian DNA) was extracted from infected tissues. Genomic DNA was extracted from all isolates using Qiagen’s DNeasy® DNA purification kit, according to the manufacturer’s instructions.

### PCR, Cloning and Sequencing

Sequences for all PCR and sequencing primers are provided as supporting information (Table S1). All of these primers were designed to be microsporidian-specific in order to avoid amplification of host DNA. A fragment of rDNA was amplified from each *N. bombycis, N. granulosis* and *V. cheracis* isolate using the primers HG4F and 5SR. The rDNA fragment was approximately 550 bp in length and contained the intergenic spacer (IGS) separating the 16S and 5S ribosomal RNA subunits. A second rDNA fragment, approximately 830 bp in length and containing the internal transcribed spacer (ITS) separating the 16S and 18S subunits was amplified from the single *V. cheracis* isolate, the Scottish *N. granulosis* isolate and the *N. bombycis* isolates from China and England using the primers ILSUF and 530R. Fragments of rDNA were also amplified from *N. apis* and *V. necatrix* isolates using the primers HG4F and HG4R. The resulting rDNA fragments were approximately 800 bp in length and contained the internal transcribed spacer (ITS) separating the 16S and 18S ribosomal RNA genes.

A fragment of the largest subunit of the housekeeping gene RNA polymerase II (*RPB1*) was amplified from each *N. granulosis* and *N. bombycis* isolate and from the isolates of *N. apis, V. cheracis, V. necatrix, V. disparis* and *N. lymantriae*. In each case, the general microsporidian primers RPB1F, RPB1R, AF1, AF3 and GR1 [46] were used to obtain preliminary sequence data. This was then used as a template to design species-specific primers. A fragment of the housekeeping gene *elongation factor-1 alpha* (*EF-1α*) was also amplified from each *N. bombycis* isolate with the primers EF1αF and EF1αR while the gene for spore *surface antigen protein 30.4* (*Sap 30.4*) was amplified from the three *N. bombycis* isolates using the primers SAPF and SAPR. Southern blot analysis indicates that *RPB1* occurs as a single copy in *Vairimorpha necatrix* [47]. Sequence similarity searches of the genomes of *N. ceranae* (the only *Nosema* genome currently available) and *E. cuniculi* (the only fully assembled microsporidian genome) were performed for *RPB1*, *EF1-α* and *Sap 30.4* using BLASTn and tBLASTx, implemented on the NCBI website with an alignment score cut-off of 200. These detected a single copy of *RPB1* and *EF1-α* in each genome. No sequences similar to *Sap 30.4* were detected in either genome, suggesting that this gene has evolved recently in the lineage containing *N. bombycis*.

All PCR products were sequenced directly in both directions using BigDye® terminators on an ABI 3100 high throughput sequencer. Chromatograms of *RPB1*, *EF-1α* and *Sap 30.4* sequences were studied carefully by eye and double-peaks indicating single nucleotide polymorphisms within a isolate were noted. This could not be accomplished for rDNA sequences because multiple indels resulted in overlapping sequences. PCR products were cloned using TOPO TA according to the manufacturer’s instructions. Plasmid DNA was purified using Qiagen’s QIAprep® Spin DNA purification kit and sequenced with primers T7 and T3. For products greater than 1 kb in length, internal primers were designed to allow full sequencing. Extensive overlap between fragments amplified by general and species-specific primer pairs ensured that most regions of DNA were amplified and sequenced at least twice, independently. This increased the likelihood of detecting PCR and sequencing artefacts.

### Analysis

Sequences were aligned using Clustal W and adjusted manually. Fasta files of sequence alignments are provided as supporting information (File Archive S1). In the cases of the rDNA spacer regions (ITS and IGS), multiple, overlapping indels rendered it impossible to align sequences with confidence in highly variable regions. Such hypervariable regions were therefore excluded from the alignments. In order to avoid cloning artifacts, differences in sequence between cloned DNA fragments were accepted as genuine polymorphisms only if they corresponded to double peaks obtained through direct sequencing. The numbers of cloned rDNA and protein-coding sequences used in the analyses are provided in Table 7.

Changes in nucleotide diversity along each rDNA sequence alignment were calculated as the average heterozygosity per site (*π*) and the average number of nucleotide differences per site (*θ_W_*) within a sliding window of 50 base pairs with an increment of 25 base pairs, implemented in Proseq 3.2 [48]. Haplotype networks were constructed for the cloned rDNA sequences obtained from *N. bombycis, N. granulosis* and *V. cheracis*, and for the *EF-1α* sequences obtained from *N. bombycis*. Connection distances between haplotypes were calculated using Arlequin [49] and the network was visualised using a force-directed algorithm, implemented in Hapstar [50].

Where several different sequences were cloned from the same gene within the same host species, recombination events were detected using RDP4 [51]. This program allows a sequence alignment to be analysed simultaneously with the RDP [52], BootScan [53], GeneConv [54], MaxChi [55], Chimaera [56], SiScan [57], 3Seq [58] and LARD [59] methods to provide a single multiple comparison (MC) Bonferroni-corrected p-value for each recombination event.

## Supporting Information

Table S1 **Sequences of PCR and sequencing primers used to produce DNA sequences.** Where these have been published previously, the reference for the original publication is provided. For all species-specific primers, a reference sequence is referred to by its GenBank accession number and the position at which the primer anneals to the reference sequence is indicated. The species-specific primers for the protein coding genes *Rpb1*, *EF-1α* and *Sap30.4* were designed using these reference sequences as templates. The use of the primer is indicated as S (sequencing only) or A/S (amplification and sequencing).
(DOCX)

File Archive S1 **Fasta files of DNA sequence alignments.**
(ZIP)
