# Supplementary material for: Diversity and Recombination of Dispersed Ribosomal DNA and Protein Coding Genes in Microsporidia
Source: PLoS One. 2013 Feb 6;8(2):e55878. doi: 10.1371/journal.pone.0055878 (PMC3566094; doi:10.1371/journal.pone.0055878)
Supplement: Table S1 — Sequences of PCR and sequencing primers used to produce DNA sequences. Where these have been published previously, the reference for the original publication is provided. For all species-specific primers, a reference sequence is referred to by its GenBank accession number and the position at which the primer anneals to the reference sequence is indicated. The species-specific primers for the protein coding genes Rpb1, EF-1α and Sap30.4 were designed using these reference sequences as templates. The use of the primer is indicated as S (sequencing only) or A/S (amplification and sequencing). (DOCX) [file pone.0055878.s001.docx]

| Species | Gene | Reference sequence | Primer | Sequence | Annealing position | Use | Reference |
| --- | --- | --- | --- | --- | --- | --- | --- |
| *N. bombycis*  *N. granulosis*  *V. cheracis* | *rRNA* | AY259631 | HG4F  5SR  ILSUF  530R | 5'-GCG GCT TAA TTT GAC TCA AC-3'  5'-TAC AGC ACC CAA CGT TCC CAA G-3'  5'-TGG GTT TAG ACC GTC GTG AG-3'  5'-CCG CGG C(T/G)G CTG GCA C-3' | 3591  4421  2350  3212 | A/S  A/S  A/S  A/S | [1]  [2]  [2]  [3] |
| *N. apis*  *V. necatrix* | *rRNA* | U97150 | HG4F  HG4R | 5'-GCG GCT TAA TTT GAC TCA AC-3'  5'-CGC CGA ATT AAA CTG AGT TG-3' | 1172  2099 | A/S  A/S | [1]  [1] |
| General | *Rpb1* |  | RPB1F  RPB1R  AF1  AF3  GR1 | 5'-CGC ACT T(C/T)G A(C/T)G G(A/C/T/G)G A(C/T)G A(A/G)G TGA-3'  5'-CCC GC(G/T) (A/G/C/T)GG (A/G/C/T)GG CAT (A/G/C/T)GC (A/G)TG (A/G)AA-3'  5'-GA(G/T) TGT CC(G/T) GG(A/T) CAT TTC GG-3'  5'- GG(A/T) CAT TTC GG(A/T) CAC ATI GA -3'  5'- TG(A/G) AA(A/C) GT(A/G) TTI AGI GTC AT(C/T) TG-3' |  | A/S  A/S  A/S  A/S  A/S | [4]  [4]  [4]  [4]  [4] |
| *N. apis* | *Rpb1* | DQ996230 | NaRPB1_1F  NaRPB1_1R  NaRPB1_2F  NaRPB1_2R  NaRPB1_3F  NaRPB1_3R | 5'-CG(A/G) AAG TGT GTG TTT TTA TTG-3'  5'-TAT TG(C/T) ATG AGG (C/T)GA TTG CT-3'  5'-TTC C(A/T)G AAG CAA TC(A/G) CCT CA-3'  5'-GCA CCG ACA GTT TTT TTG TC-3'  5'-GAT GAA GAG GAT TTA GAA AAT G-3'  5'-GTT TCT GCA GTT TTA ATA GCT GTA TC-3' | 27  783  776  1531  1456  2158 | A/S  S  S  S  S  A/S | [5]  [5]  [5]  [5]  [5]  [5] |
| *N. bombycis* | *Rpb1* | DQ996231 | NbRPB1_1F  NbRPB1_1R  NbRPB1_2F  NbRPB1_2R  NbRPB1_3F  NbRPB1_3R  NbRPB1_4F  NbRPB1_4R  NbRPB1_5F  NbRPB1_5R | 5'-GGG CAT ATC GAA CTC TCT AA-3'  5'-AGC CAC ATG AAA TTG TAA AAG-3'  5'-CCT TAA GAA ATA CGA AAT GGA-3'  5'-TCA ATT AAA CGA AAG TCT TCC-3'  5'-AAT CGA CGA CCT TCT CTT CAT-3'  5'-ACA TGA TTT CTC CTC CGC AT-3'  5'-ATT CGT CCA ATG AAA GTC GG-3'  5'-GCA AAT CCA AAT GGA ATT CTC-3'  5'-TCC GGG TCT AAA GGA TCT TT-3'  5'-GGG TCA TCT GAG TAG CAG GTT-3' | 1  571  513  1076  1035  1591  1526  2088  2017  2948 | A/S  S  S  S  S  S  S  S  S  A/S | [5]  [5]  [5]  [5]  [5]  [5]  [5]  [5]  [5]  [5] |
| *N. granulosis* | *Rpb1* | DQ996233 | NgRPB1_1F  NgRPB1_1R  NgRPB1_2F  NgRPB1_2R  NgRPB1_3F  NgRPB1_3R  NgRPB1_4F  NgRPB1_4R | 5'-ACT CTG AAT GCA CTC TGG GGA-3'  5'-TTC ATT TCA TCT CCA TCG AAA-3'  5'-AAA GAC ACA TGC AGG ATG GA-3'  5'-TAG CCT GTT GTT CTG GGC TTT-3'  5'-CGA CAA TGC TAA GAA TTC AGT AGA-3'  5'-CGA AGA CCT TAG AGA CTA AAT ATT C-3'  5'-AGC GTG TGT AGG ACA GCA GAA-3'  5'-CAC CAT CTC ATT GGG AGA ACA-3' | 15  998  853  1671  1634  2394  1871  2726 | A/S  S  S  S  S  S  S  A/S | [5]  [5]  [5]  [5]  [5]  [5]  [5]  [5] |
| *N. lymantriae*  *V. disparis* | *Rpb1* |  | NlRPB1_3R  NlRPB1_4F  NlRPB1_2F  NlRPB1_2R | 5'-TCT TTC ATG GAC ATA CCA GG-3'  5'-TGT CCA TGA AAG AGA GTT TTG-3'  5'- CCA GAC TGA AAG GCA AAG AA-3'  5'-CGT CTC CAT TTT GCA TGT GT-3' | 1834  1822  643  959 | S  S  S  S |  |
| *V. cheracis* | *Rpb1* | DQ996235 | VcRPB1_1F  VcRPB1_1R  VcRPB1_2F  VcRPB1_2R  VcRPB1_3F  VcRPB1_3R  VcRPB1_4F  VcRPB1_4R | 5'-TCC TGG AGT GCA TCT GCT TCT-3'  5'-ATT GAA GGG CGT GAT CTT CT-3'  5'-ACG CCA ACA TCT CCC TCG A-3'  5'-AAA TGT TCT CCC GAT CAT CC-3'  5'-AGG ATG ATC GGG AGA ACA TT-3'  5'-TGG TAG ATG TTC CCG TCA GC-3'  5'-TAC CAG TTC CAA TAC GGG GA-3'  5'-ACC CAA TTG AAG GCG CTT AT-3' | 13  772  706  1499  1498  2295  2310  2790 | A/S  S  S  S  S  S  S  A/S | [5]  [5]  [5]  [5]  [5]  [5]  [5]  [5] |
| *V. necatrix* | *Rpb1* | AF060234 | VnRPB1_1F  VnRPB1_1R  VnRPB1_2F  VnRPB1_2R  VnRPB1_3F  VnRPB1_3R  VnRPB1_4F  VnRPB1_4R  VnRPB1_5F  VnRPB1_5R  VnRPB1_6F  VnRPB1_6R  VnRPB1_7F  VnRPB1_7R | 5'-CCT ACG TCG TAA ATA CAA TCA-3'  5'-TGC TCT CAA CAT TCC TTC CA-3'  5'-GTT CTT TTA GTT CCT CCT CC-3'  5'-TGT TGT AAC TTT GCG GCA TG-3'  5'-ATT TGT CTT GTG TCT CGC CCT-3'  5'-TCT CTC ATT GAC ATA CCT GG-3'  5'-TGT CAA TGA GAG AGA GCT TTG-3'  5'-TGG AAA TTA CAT GGA CTA GCA-3'  5'-GCT AGT CCA TGT AAT TTC CA-3'  5'-CCA CTC ATC TTT GTC TTT ATC-3'  5'-GTG GTA TTT ACA GAC AGA TGG-3'  5'-TCG GGC TCG TTG GAC TAT AA-3'  5'-TAT AGT CCC ACG AGC CCA ACA-3'  5'-TGC TTT CCT TTT CGT TTC CT-3' | 2  768  725  1477  1419  2222  2229  2968  2969  3881  3898  4603  4541  4895 | A/S  S  S  S  S  S  S  S  S  S  S  S  S  A/S | [5]  [5]  [5]  [5]  [5]  [5]  [5]  [5]  [5]  [5]  [5]  [5]  [5]  [5] |
| *N. bombycis* | *EF-1α* | AB009600 | NbEF1a_F1  NbEF1a_R1 | 5'-AGG GAA TCT TGC TTT CCA AC -3'  5'-TAT CCT GCT GCA ACC CAG TTT-3' | 12  979 | A/S  A/S |  |
| *N. bombycis* | *Sap30.4* | AB107591 | NbSAP_F1  NbSAP_R1 | 5'-CGC CAA TAT TGA AAG GGT TG-3'  5'-GTC CAT GAA AGA AGT TGC GTT-3' | 10  725 | A/S  A/S |  |

References

1. Gatehouse HS, Malone LA (1998) The ribosomal RNA gene region of Nosema apis (microspora): DNA sequence for small and large subunit rRNA genes and evidence of a large tandem repeat unit size. Journal of Invertebrate Pathology 71: 97-105.

2. Huang WF, Tsai SJ, Lo CF, Soichi Y, Wang CH (2004) The novel organization and complete sequence of the ribosomal RNA gene of *Nosema bombycis*. Fungal Genetics and Biology 41: 473-481.

3. Baker MD, Vossbrinck CR, Becnel JJ, Andreadis TG (1998) Phylogeny of *Amblyospora* (Microsporida : Amblyosporidae) and related genera based on small subunit ribosomal DNA data: A possible example of host parasite cospeciation. Journal of Invertebrate Pathology 71: 199-206.

4. Cheney SA, Lafranchi-Tristem NJ, Bourges D, Canning EU (2001) Relationships of microsporidian genera, with emphasis on the polysporous genera, revealed by sequences of the largest subunit of RNA polymerase II (RPB1). Journal of Eukaryotic Microbiology 48: 111-117.

5. Ironside JE (2007) Multiple losses of sex within a single genus of microsporidia. Bmc Evolutionary Biology 7: 48.
